# Accumulation of risk factors associated with poor bone health in older adults

**DOI:** 10.1007/s11657-015-0250-3

**Published:** 2015-12-22

**Authors:** Jean Zhang, Karen Jameson, Avan Aihie Sayer, Sian Robinson, Cyrus Cooper, Elaine Dennison

**Affiliations:** 1 MRC Lifecourse Epidemiology Unit, University of Southampton, Southampton General Hospital, Southampton, SO16 6YD UK; 2 National Institute for Health Research Southampton Biomedical Research Centre, University of Southampton and University Hospital Southampton NHS Foundation Trust, Southampton, UK; 3 National Institute for Health Research Collaboration for Leadership in Applied Health Research and Care: Wessex, University Hospital Southampton NHS Foundation Trust, Southampton, UK; 4 NIHR Musculoskeletal Biomedical Research Unit, Nuffield Department of Orthopaedics, Rheumatology and Musculoskeletal Sciences, University of Oxford, Oxford, UK; 5 Victoria University, Wellington, New Zealand

**Keywords:** Clustering, Lifestyle, Bone mineral density, Smoking, Alcohol

## Abstract

**Summary:**

Clustering of factors linked with poor bone health is common in older adults and is associated with lower bone density and increased fracture risk in women.

**Purpose:**

Many factors are associated with bone mineral density, which in turn is strongly linked with risk of fragility fracture. We assessed how commonly clustering of risk factors occurs and related such clustering to bone mineral density in a population of older community-dwelling men and women.

**Method:**

This is a cross-sectional study with 498 men and 498 women aged 59 to 72 years, who were participants in the Hertfordshire Cohort Study, in whom incident fracture was recorded. Physical activity, diet quality, history of prior fracture, family history of fracture, cigarette and alcohol consumption and comorbidities were obtained through baseline questionnaire. Measurements of grip strength and bone mineral density of the lumbar spine and total femur were also taken.

**Results:**

Clustering of risk factors was common, with over 30 % having two or more. In women, a graded association between the number of risk factors and low bone density was seen, and strong relationships were also seen between the number of risk factors and incident fracture; women with three or more risk factors had an adjusted hazard ratio (HR) of incident fracture of 5.98 (1.67, 21.43; *p* = 0.006) compared to women with no risk factors; women with two risk factors had an adjusted HR of 2.97 (1.14, 7.74; *p* = 0.03) and those with one, 2.28 (0.90, 5.75; *p* = 0.08).

**Conclusion:**

Clustering of risk factors for poor bone health is common in community-dwelling older adults and is associated with increased risk of fracture and adverse bone health in women.

## Introduction

Certain factors are known to be associated with bone mineral density. In turn, low bone mineral density is strongly linked with fragility fracture, which is associated with a significant personal and public health burden. In the UK, one in two women and one in five men over the age of 50 years will suffer a fracture in their remaining lifetime [1]. This results in serious consequences relating to loss of mobility, independence and self-esteem [1]. UK fragility fracture cost is currently estimated at 9 million per year, and across Europe at 32 billion euros per year [2]. With an aging population across the globe, the burden is set to rise. The advent of fracture prediction tools allows us to estimate a 10-year probability of fracture, from which will follow a discussion about lifestyle modification and therapeutic intervention in some patients. We wondered how common factors associated with poor bone health are in an unselected older population and used a well-characterised UK cohort (the Hertfordshire Cohort Study) to consider this.

We chose to consider a number of factors available to us that included factors included in the fracture prediction tool FRAX. A number of lifestyle factors are well known to be associated with bone health and were available in our dataset; higher levels of physical activity have been shown to be positively associated with bone mineral density [3–7]. Smoking is commonly reported as a negative influence on bone health [3, 4], and indeed an independent and dose-dependent effect of cigarette smoking on bone loss has been documented in a meta-analysis [5]. By contrast, alcohol consumption has a varied reported effect on bone health; although modest alcohol consumption is considered beneficial, heavy intake is commonly associated with deleterious changes on bone health [6–8]. Dietary factors are also important for bone health [9, 10] and were included in our assessment despite their absence from fracture prediction tools.

We also considered other patient characteristics in this study. Specifically, we included prior personal history of fracture of family history of fracture as they are included in fracture prediction tools. Finally, we also added low grip strength to our measures of interest. There is a clear relationship between skeletal muscle and bone mass throughout the life course, and grip strength represents an easily accessible measure of low muscle mass, so it was included for this reason.

This study follows on from previous work that has demonstrated that clustering of risk factors for adverse health outcomes occurs, with a recent study highlighting the association of such clustering with poor physical performance in late adulthood; this study considered the relationship between number of lifestyle risk factors out of low physical activity, poor diet, obesity and smoking, reporting that more risk factors were associated with poorer physical function [11]. Similarly, coexistence between certain lifestyle choices (low physical activity, poor diet and smoking and increased alcohol consumption (greater than the UK recommendation)) has been shown to be associated with excess mortality [12]. Consistent with these findings, a combination of healthy lifestyle behaviours, i.e. not smoking, adopting a healthy diet and regular physical activity, appears to reduce the risk of all-cause mortality [13]. However, to our knowledge, no previous studies have considered whether some individuals display many characteristics that make them particularly vulnerable to poor bone health. Such information may be clinically useful because it would suggest that there may be a number of older individuals for whom attention is particularly warranted.

## Methods

In the late 1990s, 3000 men and women aged 59–73 years were recruited to a study, which was designed to examine the relationship between growth in infancy and the subsequent risk of adult disease, including osteoporosis (the Hertfordshire Cohort Study [14]). The selection procedure for these individuals was as follows: in brief, with the help of the National Health Service Central Registry at Southport and Hertfordshire Family Health Service Association, we traced men and women who were born during 1931–1939 in Hertfordshire and still lived there during the period 1998–2003. The birth weight and weight at 1 year of age of each individual had been recorded in a ledger by a team of midwives and health visitors who had attended each birth in Hertfordshire in the 1930s and visited the child’s home at intervals during the first year of life.

The baseline analysis is based on the 498 men and 498 women who participated in baseline clinic visits held in 1999–2003, which included baseline assessment of bone health through DXA. Participants were invited to attend for DXA based on their place of residence; as the DXA scanner was based in Hertford, East Hertfordshire residents were invited to attend, with the only exclusions being use of medications for osteoporosis (excluding hormone replacement therapy in women). Lifestyle risk factors were assessed at baseline by nurse-administered questionnaires and included information on smoking habits (current or historical, allowing calculation of pack years) and alcohol consumption (number of units consumed per week). Physical activity was assessed from responses to questions about the frequency and duration of gardening, housework, climbing stairs and carrying loads in a typical week, and included leisure activities. A standardised activity score ranging from 0 to 100 was calculated, with a score of <50 classified as low activity. The questionnaire has been designed specifically to characterise the level of physical activity in the elderly community-dwelling population in [15]. Data on history of prior fracture and participants’ comorbidities was also recorded, which included bronchitis, diabetes, ischaemic heart disease (IHD), hypertension and stroke.

Diet was assessed using a food frequency questionnaire (FFQ) [16]. Foods were categorised into 51 groups based on their type and nutrient composition. Principal component analysis of the reported weekly frequencies of consumption of the food groups was used to describe the dietary patterns of the men and women [10]. The first component described a ‘prudent’ dietary pattern that follows recommendations for a healthy diet, characterised by high consumption of fruit, vegetables, whole-grain cereals and oily fish and low consumption of white bread, chips, sugar and full-fat dairy products. A prudent diet score was calculated using the coefficient of each food group multiplied by the reported frequency of consumption of the food group, with the sum of these values providing a single score for each participant. Thus, a participant with a high prudent diet score has a high consumption of fruit, vegetables, whole-grain cereals and oily fish; participants with a low prudent diet score have high consumption of white bread, chips, sugar and full-fat dairy products. The prudent diet score was used as an index of diet quality [17].

At the clinic, a Jamar hand-held isokinetic dynamometer was used to measure the grip strength of each hand three times following a standardised protocol [18]. Low grip strength was defined as a maximum grip strength of <30 kg for men and <20 kg for women. Participants were also invited to attend for measurement of bone mineral density of the lumbar spine and total femur using a Hologic QDR 4500 dual energy x-ray absorptiometer.

**Fig. 1:**
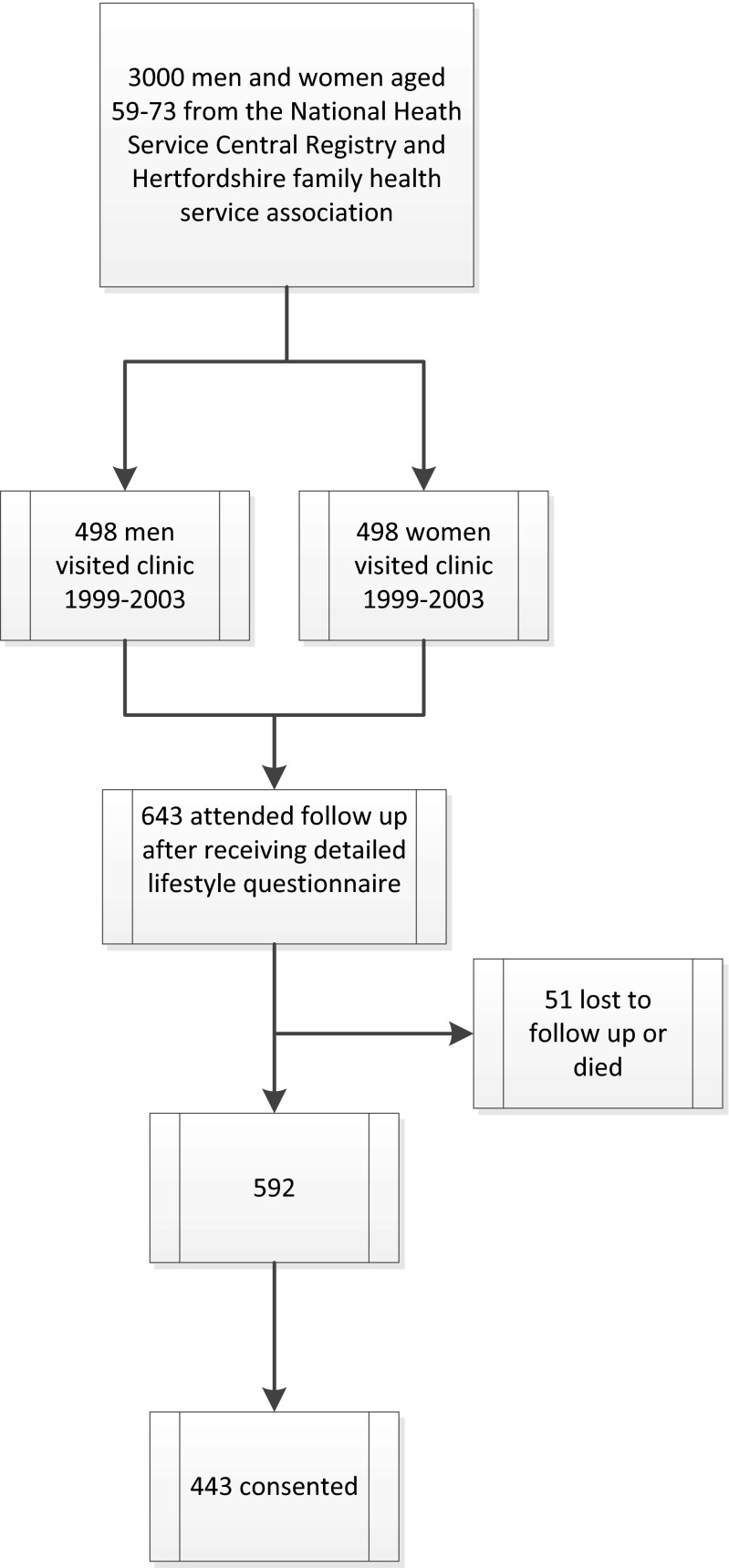
Recruitment flow chart

In 2004–2005, a subgroup of 643 attended a follow-up clinic, where a detailed lifestyle questionnaire was administered (Fig. 1). Between 2005 and 2011, 51 people became ineligible for the study as they had either moved out of Hertfordshire or had died. In March 2011, the remaining 592 men and women were approached, and 443 consented to participate in a further study. The East and North Hertfordshire Ethical Committees granted ethical approval for the study, and all participants gave written informed consent. This study was funded by the Medical Research Council and Arthritis Research UK.

### Statistical analysis

Variables were assessed for normal distribution. Risk factors were selected on the basis of measures that were readily available, and for which a reasonable literature suggested an association with bone health; we included grip strength in this panel given the emerging literature regarding bone and muscle health and prior personal or family history of fracture given its strong association with future fracture risk, even though these two measures are not ‘lifestyle’ factors but rather risk factors that might be relevant in clustering. Risk factors were then categorised to look at the effect of increasing number of lifestyle factors on bone health: low activity was defined as a physical activity score ≤50; poor diet was defined as a prudent diet score in the bottom quartile; current smoker; alcohol consumption greater than the recommended UK units per week (21 for men, 14 for women); low grip strength was defined as <30 kg for men and <20 kg for women; and previous fracture after the age of 45 and a family history of fracture after the age of 45. The number of comorbidities (bronchitis, diabetes, IHD, hypertension and stroke) was collected from self-reported data and clinic data. IHD was defined as the presence of typical angina pectoris (Rose questionnaire), angioplasty, coronary artery bypass or presence of significant Q waves on electrocardiogram. The risk factors were assessed in a binary fashion to investigate the associations between lifestyle factors and bone health. Although we considered body mass index in this panel, obesity (body mass index (BMI) >30 kg/m^2^) was associated with a higher BMD and was therefore not included in this model, although it was included in our incident fracture model as it was not associated with incident fracture on univariate analysis. Unadjusted and adjusted regression analyses were carried out looking at the clustering effect of the risk factors and its association with BMD expressed as negative *Z* scores, to demonstrate an association of more factors with worse bone outcomes. Data analysis was carried out using Stata version 13 [19].

## Results

The baseline characteristics of the participants are summarised in Table 1. Participants were community dwelling and in their seventh decade at baseline. As expected, the men overall had a higher bone mineral density (BMD) at baseline than women at the two measured sites, total femoral and lumbar spine. Men were marginally more active than women although women had a healthier diet. Again unsurprisingly, men had stronger grip strength at baseline. A greater proportion of women were categorised as never smokers (62.2 %) compared with men (33.5 %), but the percentage of current smokers were comparable in men and women (14.7 and 9.5 %, respectively). Men appeared to consume on average more alcohol than women. Women were more likely to report a family history of fracture (33.9 % compared to 20.0 %). Men and women reported similar numbers of comorbidities. The proportion of men with two or more risk factors were greater compared with women (40.7 and 30.1 %, respectively).

**Table 1:** Summary of characteristics of cohort

Characteristics	Men	Women	*p* value^c^
Age (years), *n*, mean ± SD	498, 64.8 ± 2.5	498, 66.4 ± 2.5	<0.001
BMI (kg/m^2^)	498, 26.6 ± 1.1	498, 26.9 ± 1.2	0.417
Total femoral BMD (g/cm^2^), *n*, mean ± SD	495, 1.04 ± 0.13	497, 0.90 ± 0.13	<0.001
Lumbar spine BMD (g/cm^2^), *n*, mean ± SD	497, 1.08 ± 0.16	498, 0.96 ± 0.17	<0.001
Activity score, *n*, mean ± SD	498, 64.0 ± 14.8	498, 61.2 ± 15.0	0.004
Prudent diet score, *n*, mean ± SD	498, 0.78 ± 2.06	498, 0.67 ± 1.71	<0.001
Maximum grip (kg), *n*, mean ± SD	498, 44.1 ± 7.3	498, 27.7 ± 5.1	<0.001
Alcohol consumption (units per week), *n*, median, IQR	498, 9.3, 2.5–21.5	498, 1.5,0.0–5.0	<0.001
Smoker status, *n* (%)	498 (100)	497 (100)	<0.001
Never, *n* (%)	167 (33.5)	309 (62.2)	
Ex-smoker, *n* (%)	258 (51.8)	141 (28.4)	
Current, *n* (%)	73 (14.7)	47 (9.5)	
Previous fracture after age 45, total *n*, *n* (%)	498, 63 (12.7)	498, 82 (16.5)	0.088
Family history of fracture after age 45^a^, total *n*, *n* (%)	491, 98 (20.0)	496, 168 (33.9)	<0.001
No. of comorbidities^b^, *n* (%)	471	477	0.120
0, *n* (%)	257 (54.6)	254 (53.2)	
1, *n* (%)	140 (29.7)	156 (32.7)	
2, *n* (%)	56 (11.9)	60 (12.6)	
3+, *n* (%)	18 (3.8)	7 (1.5)	
Incident fracture (after baseline), total *n*, *n* (%)	314, 23 (7.3)	318, 47 (14.8)	0.003

^a^A fracture in a parent or sibling after the age of 45

^b^Out of bronchitis, diabetes, IHD, hypertension and stroke

^c^ *p* value for difference between men and women

In men, alcohol consumption greater than the UK recommended units per week was associated with higher BMD at the total femur and the lumbar spine [−0.195 (95 % CI −0.399, 0.010), *p* value 0.062 at the total femur; −0.217 (95 % CI −0.421, −0.014), *p* value 0.036 at the lumbar spine]. In women, having previously had a fracture after the age of 45 was associated with lower BMD at both the total femur and lumbar spine [0.513 (95 % CI 0.280, 0.746), *p* value <0.001 total femur; 0.452 (95 % CI 0.218, 0.687), *p* value <0.001 lumbar spine].

**Fig. 2:**
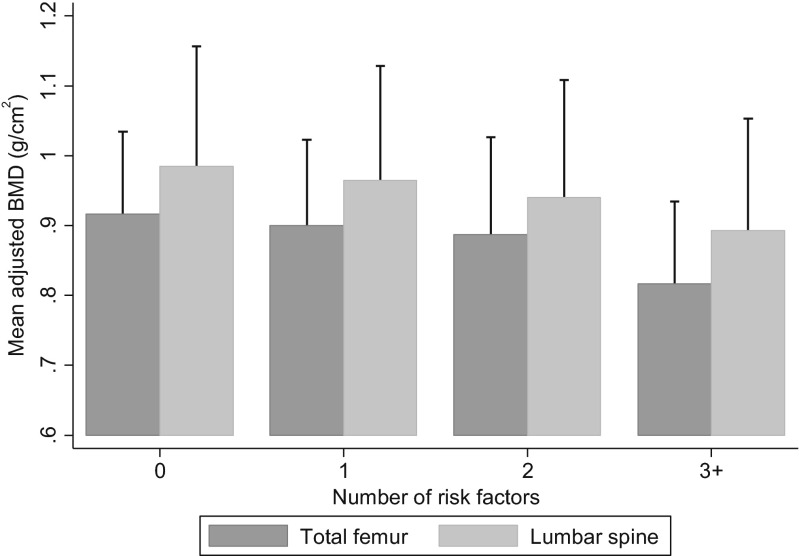
Bone mineral density in women by number of risk factors, after adjustment for age and number of comorbidities

Clustering of adverse risk factors did occur and is shown in Table 2. In our study population, over 30 % of the men and women surveyed had two or more risk factors, and these were associated with demonstrable adverse bone outcomes in women only (Table 3). There was a graded association between the number of risk factors and bone density at the lumbar spine and total femur in women (Table 3 and Fig. 2), with lowest bone densities observed in women with three or more risk factors. Results in men were non-significant (Table 3).

**Table 2:** Distribution of number of risk factors in the study population

Number of risk factors	Men		Women	
	*n*	%	*n*	%
0	96	19.6	150	30.3
1	195	39.7	196	39.6
2	144	29.3	115	23.2
3+	56	11.4	34	6.9

Number of risk factors out of low activity (activity score ≤50), poor diet (prudent diet score in bottom quartile), current smoker, alcohol consumption > recommended units (21 per week for men, 14 per week for women), low grip (<30 kg for men, <20 kg for women), previous fracture (since aged 45) and family history of fracture

**Table 3:** Graded association between number of risk factors and BMD (negative Z score) in men and women

Number of risk factors	Women						Men					
	Total femur			Lumbar spine			Total femur			Lumbar spine		
	Adjusted^a^ (*n* = 473)			Adjusted^a^ (*n* = 474)			Adjusted^a^ (*n* = 461)			Adjusted^a^ (*n* = 463)		
	Regression coefficient	95 % CI	*p* value	Regression coefficient	95 % CI	*p* value	Regression coefficient	95 % CI	*p* value	Regression coefficient	95 % CI	*p* value
0 (reference)	0	(0.000, 0.000)		0	(0.000, 0.000)		0	(0.000, 0.000)		0	(0.000, 0.000)	
1	0.129	(−0.078, 0.336)	0.223	0.115	(−0.096, 0.327)	0.285	0.072	(−0.180, 0.325)	0.575	−0.005	(−0.256, 0.246)	0.969
2	0.223	(−0.014, 0.460)	0.065	0.255	(0.013, 0.497)	0.039	−0.017	(−0.285, 0.252)	0.903	−0.010	(−0.277, 0.256)	0.939
3+	0.758	(0.392, 1.124)	<0.001	0.531	(0.157, 0.905)	0.005	0.226	(−0.110, 0.563)	0.186	0.213	(−0.121, 0.547)	0.210

^a^Adjusted for age and number of comorbidities (bronchitis, diabetes, IHD, HTN and stroke)

As detailed above, not all participants attended a follow-up visit that detailed incident fracture. Of those that did attend, more women than men reported incident fracture; 47 (14.8 %) compared with 23 (7.3 %). Among individuals in whom follow-up data were available, we observed similar relationships with incident fracture as with baseline BMD among women. Those women with three or more risk factors had an adjusted hazard ratio (HR) of incident fracture of 5.98 (1.67, 21.43; *p* = 0.006) compared with women without risk factors, while women with two risk factors had an adjusted HR of 2.97 (1.14, 7.74; *p* = 0.026) and those with one, 2.28 (0.90, 5.75; *p* = 0.081).

## Discussion

We have shown that clustering of lifestyle factors occurs and is related to poorer bone health in a cohort of elderly community-dwelling women. This negative relationship is seen with both BMD, and also with incident fracture, where women with three or more risk factors had an adjusted HR of incident fracture of 5.98 (1.67, 21.43; *p* = 0.006) compared with women without risk factors. These data highlight the importance of relevant, effective interventions in such high-risk populations. The risk factors we have selected include factors that can be readily measured in a clinical setting (with the exception of prudent diet score) and that are known to be associated with poor bone health. While some are lifestyle factors that might be modifiable by the patient, some are characteristics (low grip strength and prior fracture for example) that might be considered the result of lifestyle choice. The accumulation of these factors in some individuals is highlighted in this study, and while the association of such accumulation with adverse bone health is more clearly demonstrable in men than women, the same patterns appear in both sexes.

There are some limitations to our study; our cohort is based on a group of men and women recruited because they were born in Hertfordshire and still lived there in adult life. However, we have previously shown this group to be representative of the UK population with regard to lifestyle characteristics such as body mass index and smoking habits [14]. Furthermore, incident fracture data was not available in all subjects who attended at baseline, was collected by questionnaire and was not validated against medical records. It is likely that some relevant fractures (vertebral fractures) may be underreported because of well-known failure to recognise such fractures clinically, and in addition, there may be some recall bias when collecting data from questionnaire. Fractures occurred at low trauma (a fall from standing height or less) in all cases. Unfortunately, falls information was not available in this wave of the cohort study and could not be included in our models. The accuracy of lifestyle data will be liable to recall bias, but the similarity to national values is reassuring. Although we were unable to follow up all baseline study participants, we have included the follow-up fracture data where available as our comparisons are internal and highlight the association of several risk factors with incident fracture among women in this group.

That multiple risk factors will impact upon health is well recognised, but this study represents an opportunity to consider how common accumulation of these factors in an unselected community-dwelling older population is. We have presented the association of accumulation of risk factors with bone outcomes, but an equally important finding of this study is the demonstration that, even in a cohort study where a healthy bias might be expected to operate, over a third of our sample had at least two risk factors of poor bone health. The Hertfordshire Cohort Study has previously been demonstrated to be representative of the UK population [18], and although follow-up data might be limited in those participants with poorer baseline health, our ability to make internal comparisons in individuals for whom longitudinal data are available is still useful. In addition, while the concept of coexistence of certain lifestyle choices has been explored in other studies, the effect on bone health has not, to our knowledge, been reported in this way previously. There have been previous studies looking at effects of diet, physical activity, smoking and alcohol intake on mortality [16, 20], and others looked at how lifestyle choices impacted on the risk of developing stroke [21, 22]. Individually, many of the risk factors measured in our study have been linked to BMD negatively. The effect of smoking has been analysed to be independently linked to bone health, and a dose-dependent effect on bone loss has been made [5]. Several studies have shown the positive relationship between increased physical activity and bone health [23, 24]. BMI is another positive determinant of bone health [25, 26] and a low BMI is associated with an increased fracture risk [27]. Excessive alcohol consumption is well known to cause deleterious effect on bone health. We have linked the risk factors known to have a role in the health of bones and found that the clustering effect does occur, and the more risk factors that are present in the cluster, the greater the effect it has on bone health in women.

We have observed stronger relationships in women than in men, despite a higher prevalence of clustering of many risk factors in men. On univariate analysis, low grip strength and prior fracture were both associated with reduced BMD in women, while a high BMI was protective in both sexes. It is possible that some risk factors are stronger than others (prior fracture for example), and if more common in women, this may be relevant, as may the accelerated bone loss that occurs peri-menopausally in women. Although the trends we observed in men were not statistically significant, they appeared consistent with the patterns seen in women. However, our observations regarding clustering of risk factors in both sexes suggest that education in both men and women is important; our analysis is based on a longstanding cohort where a healthy cohort effect might be expected to operate, and so our estimates are likely to be conservative.

In summary, clustering of certain lifestyle factors occurs in both sexes and adversely affects bone health with respect to reduced bone density and increased rates of fracture in women. Health education programmes may play an important role in educating the population about their lifestyle choices and reduce these risks.
